# Exploring salivary metabolites as biomarkers in chronic craniofacial and orofacial pain: a metabolomic analysis

**DOI:** 10.1007/s11306-025-02336-x

**Published:** 2025-09-04

**Authors:** Weronika Jasinska, Yonatan Birenzweig, Yair Sharav, Doron J Aframian, Yariv Brotman, Yaron Haviv

**Affiliations:** 1 Department of Life Sciences, Ben-Gurion University of the Negev, Beer-Sheva, Israel; 2 Hirszfeld Institute of Immunology and Experimental Therapy, Polish Academy of Sciences, Wroclaw, Poland; 3 Department of Oral Medicine, Sedation and Imaging, Hadassah Medical Center, Faculty of Dental Medicine, Hebrew University of Jerusalem, Jerusalem, Israel; 4 School of Plant Sciences and Food Security, Tel Aviv University, Tel Aviv, 6997801 Israel

**Keywords:** Metabolites, Saliva, Chronic pain, Orofacial pain, Craniofacial pain, Saliva biomarker, LC-MS

## Abstract

**Introduction:**

Chronic facial pain (CFP) includes a range of conditions such as musculoskeletal, neurovascular, and neuropathic disorders affecting the facial and jaw regions, often causing significant distress to patients.

**Objectives:**

This study aims to investigate the metabolomic profile of patients with CFP, focusing on salivary metabolites as potential biomarkers for pain diagnosis and management.

**Methods:**

Metabolomics investigation was performed using combined liquid chromatography with mass spectrometry (UPLC-MS) for metabolic profiling.

**Results:**

A comprehensive analysis was conducted, utilizing both untargeted and targeted metabolomics to examine 28 metabolites previously associated with pain conditions. The results revealed significant differences in 18 metabolites between the CFP group and a control group, with seven metabolites consistently showing elevated levels regardless of gender: dl-Isoleucine, dl-Glutamine, dl-Citrulline, d-(+)-Pyroglutamic acid, dl-Tryptophan, dl-Phenylalanine, and Spermidine.

**Conclusions:**

The findings suggest a potential link between specific salivary metabolites and CFP, highlighting the complexity of pain mechanisms. Further research is needed to understand the causality and implications of these metabolic changes, which could lead to more targeted and personalized approaches in managing pain.

**Supplementary Information:**

The online version contains supplementary material available at 10.1007/s11306-025-02336-x.

## Introduction

Nociception and acute pain play an essential protective role in preventing tissue damage. When peripheral nociceptors are activated due to tissue injury, pain serves as a self-protective biological response. However, pain without a protective function is known as dysfunctional or maladaptive. This type of pain does not aid healing or prevent further harm and is considered a disease in some cases (Gustin et al., 2011). The transition from acute to chronic pain involves significant changes, known as functional plasticity, which affect molecular and anatomical networks within the nociceptive pathway (Kuner & Flor, 2017). Numerous biomarkers, often metabolites, can excite or sensitize nociceptors, and metabolomics is a powerful tool for understanding the genetic basis of metabolic variation (Misra et al., 2019). Saliva, containing small amounts of plasma, has been shown to carry genomic, transcriptomic, and proteomic markers, making it a promising diagnostic tool for detecting local and systemic conditions (Dame et al., 2015; Dewhirst et al., 2010). Identifying specific metabolite profiles in different types of pain, as compared to healthy individuals, can serve as diagnostic markers and therapeutic targets. Saliva, a less invasive and stress-free diagnostic medium, presents a potential alternative to blood or plasma. Chronic headaches and craniofacial pain are defined as conditions that occur for more than two hours per day on at least 50% of the days over a three-month period (Nicholas et al., 2019). Mechanisms such as peripheral and central sensitization have been linked to pain chronicity, explaining phenomena like hyperalgesia, allodynia, and pain referral (Sessle, 2017). Standardized protocols for defining headaches and facial pain are provided by the International Classification of Headache Disorders (ICHD) and the International Classification of Orofacial Pain (ICOP) (Benoliel et al., 2019; Levin et al., 2013). Few studies have been published to date that measure a limited number of metabolites in chronic headache and facial pain conditions. None of these studies have used saliva as a medium or compared different pain types from different sources. Few studies have measured metabolites in chronic headache and facial pain, and none have used saliva or compared various pain sources. A study by Sanches et al. (2020) found altered saliva metabolites in women with temporomandibular disorders, including increased levels of isovalerate and acetoin and decreased levels of phenyl acetate and dimethylamine (Lalue Sanches et al., 2020). Another study by Livshits et al. (2018) reported low levels of epiandrosterone sulfate (EAS) in patients with chronic masticatory myofascial pain. Chronic migraines (CM), facial migraines, and cluster headaches (CH) are classified as primary headaches according to the ICHD-3. Metabolites linked to these disorders have been examined in several studies. *Curto et al. (2016a) found abnormalities in the kynurenine pathway in patients with CM and CH, including reduced concentrations of L-kynurenine, kynurenic acid, and other metabolites (Davis and Liu, 2015; Curto et al., 2016a, b). D’Andrea et al. (2013) also measured lower tryptamine (TRY) levels in CM patients but higher TRY in chronic CH patients. Orofacial neuropathic pain, caused by nerve damage or dysfunction, includes trigeminal neuralgia, post-traumatic neuropathy, and burning mouth syndrome (BMS). Studies have linked these conditions to altered metabolite profiles, such as decreased L-dopa and L-tyrosine in BMS patients (Moreau et al., 2023). Alexander et al. (2013) also observed increased glutamate and aspartate levels in patients with complex regional pain syndrome (CRPS), highlighting the role of glutamate receptors in hyperalgesia. Metabolomics provides valuable insights into disease-specific metabolic changes, offering potential biomarkers for early diagnosis and treatment monitoring. Since metabolites undergo more significant changes than genes or proteins, they are powerful indicators of the body’s response to disease and treatment (López-López et al., 2018). Given that most research on orofacial pain has focused on blood-based metabolomics, our study aims to expand salivary metabolomics profiling to improve understanding of chronic headaches and craniofacial pain. We propose conducting both targeted and untargeted metabolomics analyses on the saliva of patients with chronic facial pain and headaches. The targeted approach will focus on metabolites previously linked to pain, while the untargeted approach will provide a broader understanding of metabolomic profiles. This research aims to advance personalized treatment strategies for craniofacial pain.

## Materials and methods

### Participants and collection of medical records

The study adhered to the STROBE guidelines and received approval from the Hadassah Medical Organization’s (HMO) Institutional Review Board (IRB) (Approval No. 0662-HMO-17). All data were fully anonymized. Written informed consent was obtained from all participants, including both pain patients and control subjects, in accordance with IRB guidelines. Medical records of 100 participants who visited the Orofacial Pain Clinic at the Hebrew University-Hadassah Faculty of Dental Medicine between 2017 and 2018 were reviewed. Among them, 63 were clinic patients, while 37 (21 females, 16 males) served as age-matched pain-free controls.

- **Inclusion Criteria**:
  - Age over 18 years.
  - Diagnosis of chronic facial pain (CFP) for at least 3 months.
- **Exclusion Criteria**:
  - Refusal/inability to consent.
  - Medical conditions affecting salivary gland function (e.g., autoimmune diseases like Sjögren’s syndrome, rheumatoid arthritis).
  - History of head and neck cancer treated with radiotherapy, chemotherapy, or biologically targeted therapies.
  - Graft-versus-host disease.
  - Fibromyalgia.
  - Insufficient saliva sample (less than 0.2 ml in 10 min).

Collected medical records included diagnosis, demographics (gender, age, BMI), medication, and relevant medical history. Pain onset, intensity, and quality were also documented.

### Craniofacial pain diagnosis

Based on the diagnosis, etiology, and pain characteristics of chronic facial pain (CFP), the patients were divided into three groups as described by Sharav and Benoliel (Haviv et al., 2015):

- **Musculoskeletal group**: This group included patients with pain originating from temporomandibular disorders (TMD), such as masticatory muscle pain (MMP), temporomandibular joint (TMJ) pain, and combined muscle and joint pain. Diagnoses were made according to the Diagnostic Criteria for Temporomandibular Disorders (DC/TMD) (Schiffman et al., 2014).
- **Neurovascular group**: Although chronic tension-type headache is primarily considered a musculoskeletal disorder, some individuals may experience additional neurovascular symptoms or underlying pathophysiology. Both migraine and tension-type headaches were diagnosed according to the ICHD-3 criteria (Arnold, 2018). Additionally, “neurovascular orofacial pain (NVOP)” was used for facial pain with migrainous features in the second and/or third divisions of the trigeminal nerve (Benoliel et al., 2010).
- **Neuropathic group**: This group included patients with trigeminal neuralgia (TN) (Haviv et al., 2016), painful post-traumatic trigeminal neuropathy (PTN) (Haviv et al., 2014) and burning mouth syndrome (BMS) (Benoliel & Gaul, 2017). A thorough extraoral examination included cranial nerve assessment and palpation of the masticatory apparatus (Benoliel et al., 1994; Haviv et al., 2015). An intraoral examination was performed to exclude dental, periodontal, and mucosal pathology. For patients with TN, brain and brainstem imaging was conducted to exclude intracranial pathology.

### Saliva collection

Unstimulated saliva was collected for 10 min into pre-calibrated tubes according to established protocols (Aframian et al., 2006; Krief et al., 2019). Saliva collection occurred between 9:00 AM and 12:00 PM in order to minimize diurnal variability, we acknowledge that some metabolites may still be subject to circadian influence, which could contribute to inter-individual differences and should be considered in future studies. Participants refrained from exercising, eating, drinking, and brushing their teeth for 2 h before saliva collection. Additionally, patients did not take their medication, including sialagogues, prior to collection in order to minimize variability as much as possible. Volunteers rested for 10 min before saliva collection, sitting upright in a quiet room, and were instructed not to speak or leave the room until after the saliva was collected. Saliva samples were immediately stored at − 80 °C until further processing. In the laboratory of Prof. Yariv Brotman at the Department of Life Sciences, Ben-Gurion University of the Negev, Beersheba, samples were defrosted and centrifuged at 3500×*g* for 10 min at 2 °C to remove insoluble material, cell debris, and food remnants. The supernatant was aliquoted into polypropylene tubes and stored at − 80 °C until further analysis. Saliva samples were analyzed in the following sequence: 2 CFP samples from the same groups, followed by a gender- and BMI-matched control sample. The saliva samples were prepared and analyzed by liquid chromatography-mass spectrometry (LC-MS) for metabolite identification and quantification. The extraction of polar metabolites was performed as previously described (Lapidot-Cohen et al., 2020). Samples dried down in the SpeedVac and resuspended in 120 µl of 80% methanol. LC–MS data were obtained using a Waters Acquity UPLC system (Waters, http://www.waters.com), coupled to an Exactive mass spectrometer (Thermo Fisher, http://www.thermofisher.com). Instrumental settings were previously described (Giavalisco et al., 2011). A HSS T3 C18 reversed-phase column (100 mm × 2.1 mm × 1.8 μm particles; Waters) was used and the temperature was set to 40 °C. The mobile phases were 0.1% formic acid in H2O (Buffer A, ULC MS grade; BioSolve) and 0.1% formic acid in acetonitrile (Buffer B, ULC MS grade; BioSolve). A 5-µl sample was injected. The spectra were recorded alternating between full-scan and all-ion fragmentation-scan modes, covering a mass range from 100 to 1500 m/z. The resolution was set to 25,000, with maximum time scan 250 ms. Data extraction and analysis were conducted using Xcalibur™ Software and Compound Discoverer 3.3 (Thermo Fisher Scientific). The further processing of the MS data included isotope clustering, adduct detection, and library search. The study did not include technical replicates (i.e., repeated LC-MS injections of the same extract) or biological replicates from the same subject collected at different times. Workflow diagram summarizing untargeted vs. targeted analyses is available as Supplementary figure. Data were submitted to Metabolights website: https://www.ebi.ac.uk/metabolights/MTBLS12704.

### Statistics and data visualization

To examine the differences in salivary metabolites levels for nominal and categorical background variables, a t-test and one-way analysis of variance were performed. The differences between salivary metabolites and specific background variables were analyzed for each diagnosis separately by using the non-parametric Mann–Whitney test. Figures and *P* values were obtained after calculations from MetaboAnalyst 6.0 (https://new.metaboanalyst.ca/) and software GraphPad Prism 9.0.0 (Dotmatics).

## Results and discussions

**Fig. 1:**
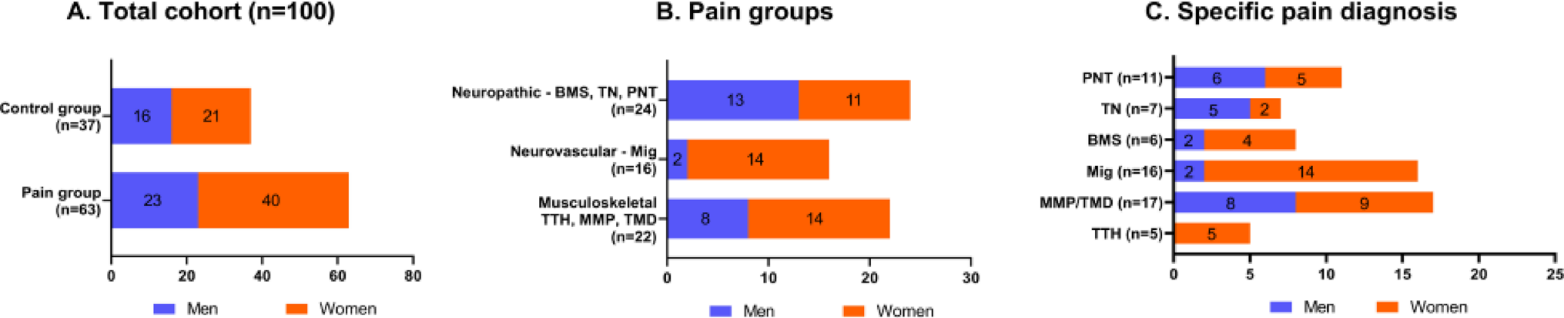
**A–C** Cohort distribution according to diagnosis and gender. Diagnoses are grouped according to etiology and characteristic, see Sharav and Benoliel (Haviv et al., 2015): *TTH* tension type headache (primary headache), *MMP* masticatory myofascial pain, *TMD* temporomandibular disorder, *Mig* migraine, *BMS* burning mouth syndrome, *TN* trigeminal neuralgia, *PTN* painful peripheral post traumatic neuropathy. In the Supplementary Table 6 statistical analysis was provided for the demographic and clinical variables (age, gender, BMI)

**Fig. 2:**
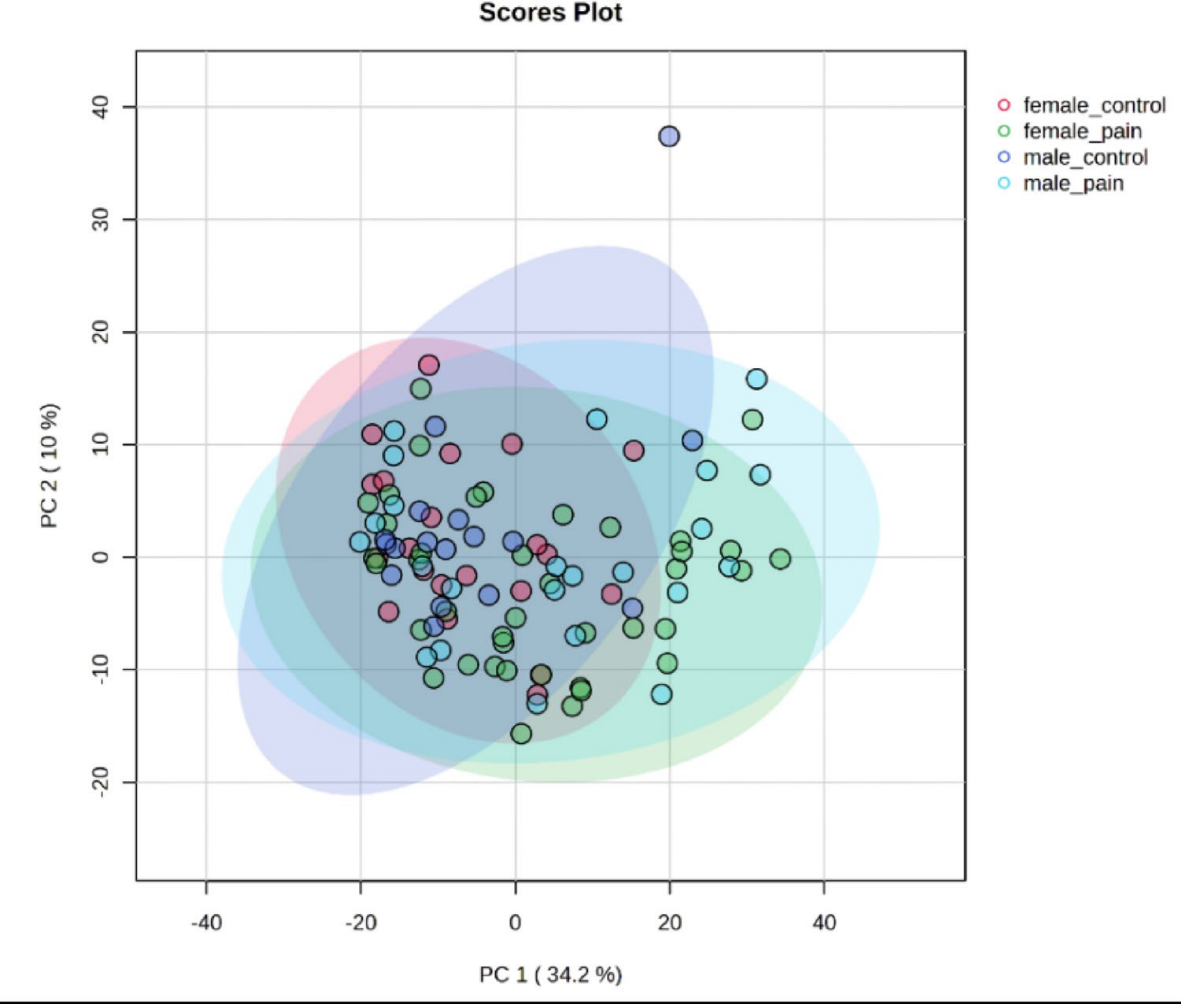
Principal component analysis (PCA) for the pain patients and controls (males and females). It shows high similarity between males and females and their response to pain. Raw data available in Supplementary Table 2

The total cohort included 100 individuals of which 63 (23 males, 40 females) were chronic craniofacial pain patients, having experienced pain more than 3 months, and 37 (16 males, 21 females) were gender- and aged-matched pain-free controls (Fig. 1A). Among the 63 chronic CFP patients, 22 (34.9%; 8 males, 14 females) were categorized as musculoskeletal group, 16 (25.3%; 2 males, 14 female) were categorized as the neurovascular group, and 24 (38.1%; 13 males, 11 females) as the neuropathic group (Fig. 2B). The specific pain diagnoses—Tension Type Headache (TTH), Masticatory Myofascial Pain (MMP), Migraine, Burning Mouth Syndrome (BMS), Trigeminal Neuralgia (TN), and Post traumatic Neuropathy (PTN)—according to gender are summarized in Fig. 1C.

### Untargeted analysis

**Fig. 3:**
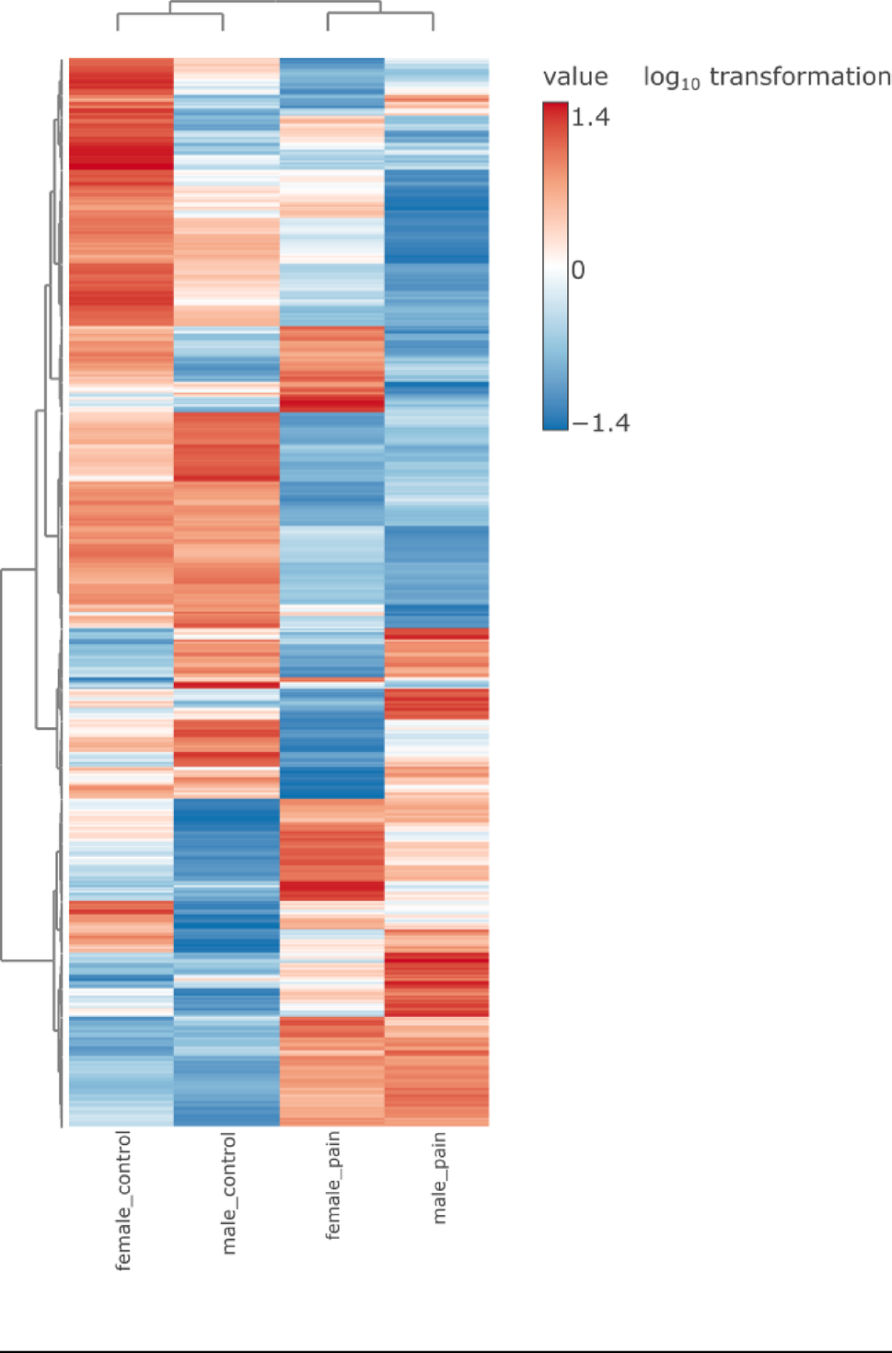
Heatmap presents changes in metabolite intensities for 4 groups of patients with a gender specification. Female and male patients with a pain experience show similar pattern between each other as well as control groups. Metabolite intensities with a log_10_ transformation. Raw data available in Supplementary Table 2

More than 2300 metabolites were detected (Supplementary Table 2). We compared patients without specifying the gender from the pain-free control group with patients experiencing pain. 861 metabolites were significantly different between the two groups (Supplementary Table 3), while 1452 metabolites were not significant. *P* values were obtained by the two-sample t-test. The next step was to compare control and pain groups with specification of the gender and visualize results by PCA (Fig. 2) and heatmap (Fig. 3). Analysis revealed high similarity between the female and male control groups and high similarity between female and male patients with pain, showing a separation between control and pain groups. Although there were differences between male and female in terms of metabolic signature, their response to pain was similar. To explore sex-specific metabolic differences, we performed a sex-stratified analysis comparing control vs. pain groups in women and men separately. Volcano plots for each sex are presented in Supplementary Fig. 2. Several metabolites (listed in Supplementary Table 5) showed significant differences only in one sex, highlighting potential sex-specific metabolic alterations associated with pain.

### Targeted analysis

The targeted analysis disclosed 28 metabolites (Table 1, Supplementary Table 4) that are known and previously described in the literature to be associated with different types of pain (Aroke et al., 2020; Livshits et al., 2018; Miller et al., 2020; Teckchandani et al., 2021).

Among the 28 annotated metabolites, 21 metabolites (75%) (marked with a star symbol next to them) showed significant differences to pain in all/ some criteria (gender-related, pain subgroups, pain types, pain characteristic continuous/attack), elaborated below.

**Table 1:** The 28 metabolites associated with pain, of which 21 metabolites significantly linked (*P* value < 0.05) to CFP in this study are star-marked

dl-Asparagine*	dl-Citrulline*
dl-Aspartic acid*	l-Carnitine
L-(+)-Arginine	4-Hydroxybenzaldehyde*
dl-Glutamic acid*	Acetyl-l-carnitine
dl-Glutamine*	Adenine*
dl-Isoleucine*	N-Acetyl phenylalanine*
dl-Ornithine*	l-Histidine
dl-Phenylalanine*	N-Acetylneuraminic acid*
dl-Proline*	Nicotinamide
d-(+)-Pyroglutamic acid*	Pipecolic acid
dl-Tyrosine*	Spermidine*
dl-Valine*	Theobromine*
dl-Tryptophan*	Uric acid
N-Acetyl-L-aspartic acid*	Urocanic acid*

### Significant metabolites specific to pain

**Fig. 4:**
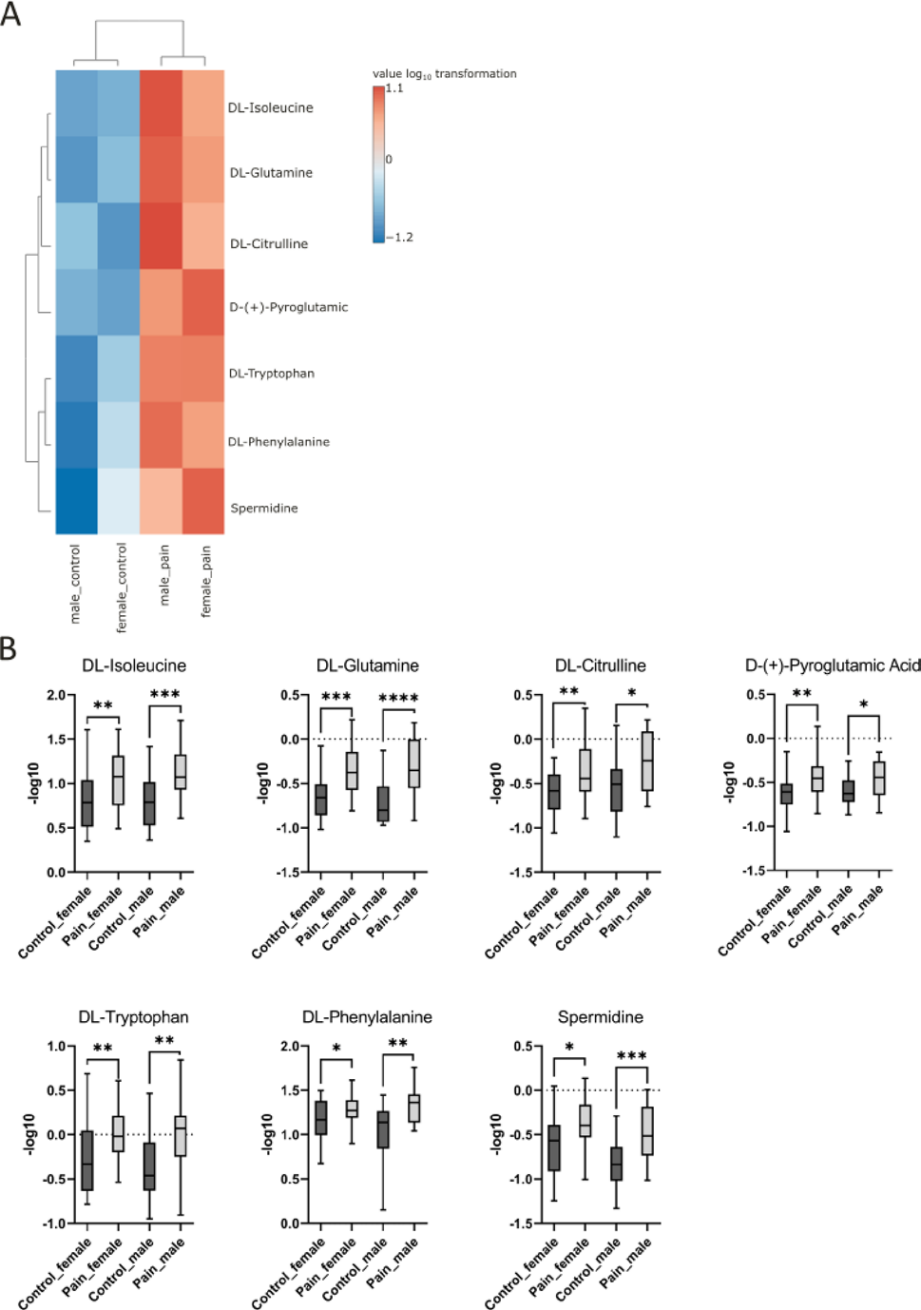
**A** Heatmap presenting 7 metabolites significantly higher in the pain group compared to the control group for both female and male, showing irrelevance to gender. Each colored cell on the map corresponds to metabolite intensity with a log_10_ transformation. **B** Boxplots presenting 7 metabolites significantly higher in the pain group compared to the control group for both female and male, showing irrelevance to gender. dl-Isoleucine [*p* = 0.005 (female); *p* = 0.0009 (male)]; dl-Glutamine [*p* = 0.0001 (female); *p* < 0.0001 (male)]; dl-Citrulline [*p* = 0.003 (female); *p* = 0.0107 (male)]; d-(+)-Pyroglutamic acid [*p* = 0.007 (female); *p* = 0.042 (male)]; dl-Tryptophan [*p* = 0.0052 (female); *p* = 0.0048 (male); dl-Phenylalanine [*p* = 0.0322 (female); *p* = 0.0063 (male)]; Spermidine [*p* = 0.0117 (female); *p* = 0.0005 (male)]

Seven significant metabolites were detected at a higher level in the pain groups as compared to the control. They were irrelevant to gender and considered specific to pain (Fig. 4).

### Gender-related metabolites

**Fig. 5:**
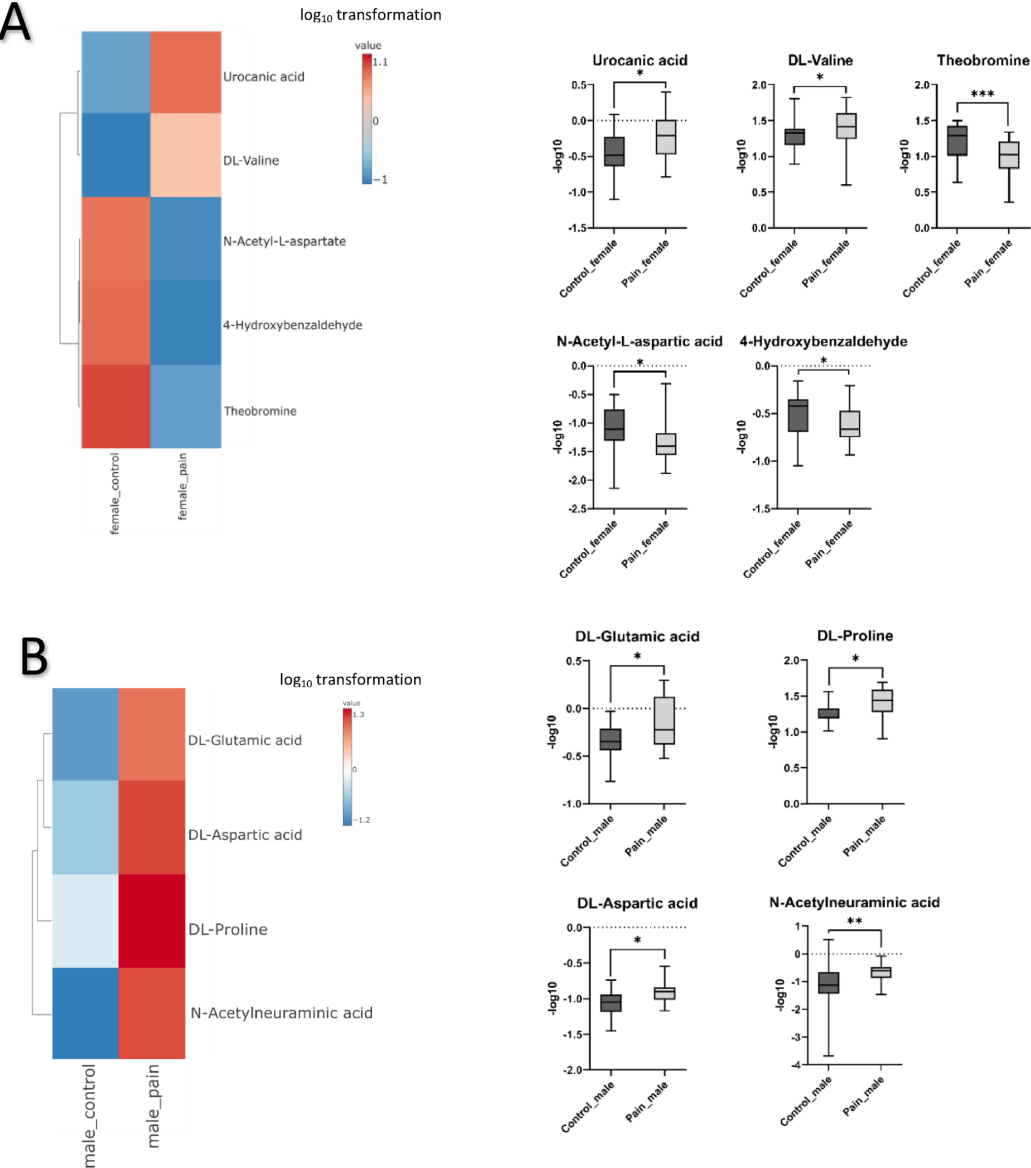
Gender-related metabolites presented in the heat-map and boxplots associate with pain group vs. control. Each colored cell on the map corresponds to metabolite intensity with a log_10_ transformation. Boxplots of significant metabolites specific to pain and gender-related. Female (**A**); male (**B**). Urocanic acid [*p* = 0.014]; dl-valine [*p* = 0.0334]; theobromine [*p* = 0.0006]; [*p* = 0.0117]; N-acetyl-L-aspartate [*p* = 0.0153]; 4-hydroxybenzaldehyde [*p* = 0.0117]; dl-glutamic acid [*p* = 0.048]; dl-proline [*p* = 0.0148]; aspartate [*p* = 0.0161]; N-acetylneuramic acid [*p* = 0.0098]

Gender-related metabolites were also found to be associated with pain (Fig. 5). For example, N-acetylneuraminic acid, dl-glutamic acid, dl-aspartic acid, and dl-proline were significantly higher (*p* < 0.05) in the male pain group vs. male control. Metabolites such as N-acetyl-L-aspartic acid, theobromine, urocanic acid, dl-valine, and 4-hydroxybenzaldehyde were significantly lower (*p* < 0.05) in the female pain group vs. female control, while urocanic acid and dl-valine were significantly higher (*p* < 0.05).

During the examination patients were asked about the type of pain (attacks or continuous pain) (Supplementary Table 1). Nine of the annotated metabolites were significantly different for attack/continuous pain vs. control group. These metabolites were: d-(+)-pyroglutamic acid, dl-aspartic acid, dl-citrulline, dl-glutamic acid, dl-glutamine, dl-isoleucine, dl-phenylalanine, dl-tryptophan, and spermidine. Additionally, comparison between attacks vs. control group revealed two other metabolites of significance difference: adenine and dl-tyrosine. Likewise, comparison between continuous pain vs. control is correlated with changes in levels of 4-hydroxybenzaldehyde, dl-asparagine, dl-ornithine, dl-proline, dl-valine, N-acetyl-L-aspartic acid, theobromine and urocanic acid. List of metabolites and *P* values is presented in Table 2.

**Table 2:** List of annotated metabolites significantly different for attack/continuous pain vs. control group, with *P* values

Attacks/continuous pain vs. control	*P* value
d-(+)-Pyroglutamic acid	< 0.003
dl-Aspartic acid	< 0.015
dl-Citrulline	< 0.011
dl-Glutamic acid	< 0.0072
dl-Isoleucine	< 0.00004
dl-Phenylalanine	< 0.009
dl-Tryptophan	< 0.0012
Spermidine	< 0.0014
Attacks vs. control	
Adenine	0.010491
dl-Tyrosine	0.026302
Continuous pain vs. control	
4-Hydroxybenzaldehyde	0.0094552
dl-Asparagine	0.048468
dl-Ornithine	0.0070655
dl-Proline	0.019276
dl-Valine	0.033082
N-Acetyl-l-aspartic acid	0.0018732
Theobromine	0.00016346
Urocanic acid	0.00046963

### Interpretation of the LC-MS metabolite profile data

Metabolomics is the study of small molecules, or metabolites, present in biological samples, which can provide valuable insights into the metabolic state of an organism. This field has the potential to identify biomarkers for diseases and monitor disease progression. Although many small molecules remain uncharacterized, metabolomics and proteomics dysregulation have been implicated in numerous chronic diseases, including cancer, neurodegenerative diseases such as Alzheimer’s and Parkinson’s, pain, periodontal diseases, and others (Gardner et al., 2020; López-López et al., 2018; Roi et al., 2019).

Saliva and plasma are both commonly used for metabolomics studies, but saliva is easier to collect since it is non-invasive (Gardner et al., 2020). Although most compounds found in blood also appear in saliva, their concentrations are lower, as they pass through passive or active transport and extracellular filtration (Dame et al., 2015). The non-invasive nature of saliva collection offers several benefits: it’s more comfortable for participants, carries fewer risks, requires less training, and can be self-collected, making it ideal for large-scale, long-term studies. Although saliva is increasingly recognized as a promising diagnostic fluid due to its non-invasive and stress-free collection, its use in chronic pain research remains limited. This is largely due to challenges such as low metabolite concentration, high variability in salivary flow, and the lack of standardized collection and analytical protocols. Saliva in general has been less studied than urine and plasma, and data on salivary metabolites related to headaches and CFP is very rare. However, saliva has been less studied than urine and plasma, and data on salivary metabolites related to headaches and chronic facial pain (CFP) is limited.

Twenty-eight metabolites were preselected before conducting statistical analyses. This selection was based on an extensive literature review, encompassing all metabolites previously identified as associated with any form of pain, including headaches and general pain (Aroke et al., 2020; Livshits et al., 2018; Miller et al., 2020; Teckchandani et al., 2021). These metabolites were chosen as part of a targeted metabolic analysis, complemented by a general untargeted analysis. Both targeted and untargeted metabolites were investigated in our study to identify significant salivary changes correlated with CFP. Our study is pioneering and unique in investigating, for the first time, salivary metabolites specific to chronic headaches and various types of CFP.

Among the 28 targeted metabolites analyzed, 18 (64.3%) showed significant changes in salivary levels in chronic CFP patients compared to controls for the first time, as they had not been specifically mentioned in the literature for facial pains. These metabolites include theobromine, N-acetylneuraminic acid, spermidine, dl-phenylalanine, dl-tryptophan, dl-isoleucine, dl-glutamic acid, dl-glutamine, urocanic acid, d-(+)-pyroglutamic acid, adenine, dl-valine, dl-aspartic acid, dl-citrulline, dl-proline, N-acetyl phenylalanine, 4-hydroxybenzaldehyde, and uric acid. Seven of the previously listed metabolites were found to be significantly higher in the pain group without specification of the gender (Fig. 4). Another 11 metabolites from the targeted analysis were found to be significantly different for the pain group vs. control group and correlated to gender (Fig. 5).

Several pain and patient characteristics were measured regarding these metabolites. The major findings beside gender were the connection to the pattern of pain: pain that comes in attacks (episodic nature); continuous everyday pain; and general pain types (Supplementary Table 1). In addition to the targeted metabolomics, an untargeted metabolomics analysis was performed, revealing more than 2,300 metabolites connected to CFP. A statistical t-test analysis identified 861 metabolites that were significantly different between patients with pain and the pain-free control group (Figs. 2 and 3). These metabolites are recommended for further study.

When comparing our study results to other published studies in the literature, the following five metabolites— dl-glutamic acid, dl-aspartic acid, dl-citrulline, and spermidine—were found to be significantly correlated with CFP pain in published studies. However, those studies focused on serum or plasma analyses (Aczél et al., 2021; Alexander et al., 2013; D’Andrea et al., 2013, 2014, 2017, 2019, 2022; Lionetto et al., 2021; Mäntyselkä et al., 2016).

Some of the metabolites can be associated with metabolic pathways and their relation to pain mechanisms. For instance, pyroglutamic acid is derived from glutathione, a vital antioxidant that helps protect cells from oxidative stress. Depletion of glutathione renders nerve cells susceptible to oxidative damage and free radicals, which can contribute to pain. High levels of pyroglutamic acid may indicate glutathione depletion, potentially contributing to oxidative stress and inflammatory processes associated with pain (Gunn, 2020).

Urocanic acid levels were higher in the female pain group. This metabolite is converted by the enzyme urocanase into glutamic acid. While both the central nervous system (CNS) and liver utilize glutamic acid, their roles and regulatory mechanisms differ significantly. In the CNS, glutamic acid is crucial for neurotransmitter metabolism and pain modulation. The elevated levels of urocanic acid in plasma—and consequently in saliva, as observed in our study—indicate increased activity of urocanase in the liver and enhanced histidine metabolism. Although histidine metabolism is not typically considered a primary factor in pain-related conditions, the elevated urocanic acid levels suggest a potential link to the metabolic processes involved in pain.

Glutamine, proline, isoleucine, and valine are essential amino acids crucial for many body functions. All were significantly higher in the pain group compared to the control group (Figs. 4, 5). A study by Smolenska et al. (2016) found a significant correlation between high plasma levels of glutamic acid (a derivative of glutamine), proline, and aspartic acid (a derivative of asparagine) in patients with rheumatoid arthritis (RA) (Smolenska et al., 2016). Furthermore, research by Albaugh et al. (2017) highlighted proline’s vital role in collagen biosynthesis and wound repair, suggesting that elevated concentrations may indicate a tissue damage repair mechanism (Albaugh et al., 2017). This suggests that the connection may not be directly related to inflammation or tissue damage (as seen in RA), but rather mediated by pain and pain-related mechanisms in the brain. Alternatively, it could indicate that chronic headaches and facial pain, previously not associated with inflammation, may indeed have inflammatory markers. However, it’s important to note that inflammatory markers were not measured; this remains a hypothesis-generating observation that may suggest a possible link between certain metabolomic changes such as those in the arginine or glutamate pathways and broader inflammatory processes in chronic pain.

Phenylalanine and tryptophan (TRP), specifically dl-phenylalanine and dl-TRP, were significantly elevated in the pain group compared to the control group (Fig. 4). Elevated plasma levels of these amino acids can impact the metabolism of various neurotransmitters in the CNS, particularly those derived from phenylalanine, such as dopamine, norepinephrine, and serotonin, and those derived from TRP, such as serotonin—all of which are involved in pain perception. While there is limited research on the specific effect of phenylalanine on chronic pain, some evidence suggests a potential role when levels are significantly high, particularly in individuals with rheumatoid arthritis (RA) (Aroke et al., 2020). Regarding TRP, some studies indicate that metabolites from the TRP-kynurenine pathway play a role in chronic pain (Tanaka et al., 2021).

N-acetylneuraminic acid (NANA) was significantly higher in the male pain group compared to the male control group only. NANA is a naturally occurring sialic acid involved in many biological processes, including inflammation. It is found in glycoproteins on cell surfaces, where it serves as an immune and inflammatory mediator, potentially impacting pain pathways. Changes in NANA levels have been observed in certain inflammatory conditions such as RA and autoimmune diseases (Mahajan & Pillai, 2016). However, currently there is no direct evidence linking NANA to chronic pain.

d-Pyroglutamic acid, dl-glutamic acid, and dl-glutamine were significantly higher in the pain group (Figs. 4, 5). Pyroglutamic acid is a derivative of glutamic acid. Elevated plasma levels of glutamic acid (and consequently salivary levels, as observed in our study) can influence glutamate and norepinephrine levels in the CNS. These neurotransmitters play a role in central sensitization, which amplifies pain and contributes to the development and maintenance of chronic pain. Glutamine is involved in neurotransmitter metabolism, including the synthesis of both glutamate and GABA (Sedlak et al., 2019).

Increased plasma levels of TRP (Fig. 4) can potentially enhance serotonin production and release in the CNS. Serotonin, in turn, can affect the release and function of norepinephrine, substance P, endorphins, adenosine, and cannabinoids—all neuromodulators that can influence pain (Ogedengbe et al., 2015). Similarly, increased plasma levels of phenylalanine (Fig. 4) may influence dopamine production and release in the CNS (Aroke et al., 2020).

dl-Citrulline, dl-ornithine, and spermidine (Fig. 4) are all essential components of the arginine pathway and were highly significant in the pain group in our study. Currently, no drugs specifically target the arginine pathway, but some drugs indirectly affect it and have been used to treat chronic pain (D'Amico et al., 2002; Rath et al., 2014; Enthoven et al., 2017). A study by Mäntyselkä et al. (2016) found similar results to ours, with significantly high serum levels of ornithine in patients suffering from chronic widespread musculoskeletal pain (Mäntyselkä et al., 2016). Mäntyselkä hypothesized that since ornithine is essential for collagen synthesis and wound repair, its elevated concentration may indicate a repair mechanism for tissue damage. Two studies by Aczél et al. (2021) and Lionetto et al. (2021) measured plasma and serum metabolites in patients suffering from chronic migraine (Aczél et al., 2021; Lionetto et al., 2021). Both studies reported a significant increase in spermidine metabolite levels. The authors hypothesized that spermidine (and spermine, another elevated metabolite) can regulate glutamate receptors, which may explain their involvement in migraine.

Additionally, elevated levels of citrulline, a marker often associated with metabolic diseases such as urea cycle disorders like citrullinemia, raise questions about the urea cycle’s activity in these patients. The presence of other urea cycle metabolites, such as ornithine, suggests either an overactive urea cycle or partial inhibition. Elevated citrulline levels have also been linked to mitochondrial dysfunction, as seen in conditions like pyruvate dehydrogenase E3 deficiency and pyruvate carboxylase deficiency. This suggests a potential mitochondrial dysfunction in chronic pain patients.

The relationship between metabolites and pain is complex because pain and metabolic processes are intricately linked and can affect each other in multiple ways (Packiasabapathy and Sadhasivam, 2018; Teckchandani et al., 2021). It is challenging to determine whether an increase in salivary or plasma metabolites leads to pain elevation or if pain initiates first, causing increased metabolite levels. For example, pain can trigger stress responses, releasing hormones like cortisol, which can affect metabolic processes and alter metabolite levels. Conversely, certain metabolites, such as inflammatory mediators and neuropeptides, can sensitize pain receptors and contribute to the development and persistence of pain (Hannibal & Bishop, 2014). Moreover, the relationship between plasma/salivary metabolites and CNS neurotransmitters/neuromodulators, which are major components in pain perception, is complex and can be influenced by various factors and regulatory systems. The effect of these metabolites can also be modulated by factors such as the blood–brain barrier (BBB) and specific receptor interactions.

Limitations: Human studies include many factors that may influence our study results, some of which are quantifiable and others seemingly impossible to measure, such as stress, sleep disorders (Kaneita et al., 2006), obesity (Chin et al., 2020; Okifuji & Hare, 2015), chronic periodontitis, and caries (Gardner et al., 2020; Pereira et al., 2020). Regarding published salivary metabolomics reviews, there is considerable variation in sample collection and preparation methods, analytical platforms, and statistical methods. This heterogeneity makes comparisons across different studies challenging.

## Conclusions

The study included 100 participants: 63 chronic craniofacial pain patients (23 males, 40 females) and 37 pain-free controls (16 males, 21 females), matched for age and sex. Pain patients were classified into musculoskeletal, neurovascular, and neuropathic subgroups, reflecting common diagnoses such as TTH, MMP, migraine, BMS, TN, and PTN. We applied several statistical tests to underline significant metabolites: t-test, non-parametric Mann–Whitney test and one-way analysis of variance. The primary significance of this paper lies at three distinct yet interconnected levels: (1) For the first time, a comprehensive series of 28 metabolites previously mentioned in the context of pain were tested in the saliva of chronic pain patients, in addition to the more commonly tested media such as plasma and CSF. (2) This study is unique in its focus on pain in the facial and jaw regions, with limited consideration of general pain conditions like rheumatoid arthritis (RA) or fibromyalgia. (3) The results revealed significant differences in 18 out of the 28 metabolites tested in saliva between the pain group and the control group. Among these, 7 metabolites were statistically elevated in the pain group. It is important to recognize that salivary metabolite levels may not directly mirror the metabolic state of specific organs or tissues implicated in chronic pain conditions. Consequently, metabolites identified in saliva should primarily be interpreted as biomarkers associated with chronic pain rather than direct evidence of organ-specific metabolic dysfunction. However, this finding is of interest, as it suggests a possible link between pain and the balance of specific metabolites in the body at times appearing more prominent than differences related to gender. Nonetheless, this observation should be interpreted cautiously. We reviewed some of the metabolites and their possible connections to pain, presenting literature-based hypotheses. However, the findings are correlative and should not be interpreted as evidence of causality. These associations are hypothesis-generating and require further validation through integrated pathway-level analyses and multi-omics approaches. This finding is noteworthy as it suggests a potential link between pain and the specific balance of these metabolites in the body, even surpassing the influence of the patient’s gender. As part of the discussion, we reviewed some of the metabolites and their possible connections to pain, presenting literature-based hypotheses. However, due to the complexity and multitude of potential factors, this general article cannot conclusively determine the role of each metabolite in pain. It remains unclear whether the elevated metabolites in chronic pain patients are a cause of pain development or persistence, or a result of chronic pain and its effects on the body.

## Supplementary Information

Below is the link to the electronic supplementary material.

Supplementary Material 1 (178 KB)

Supplementary Material 2 (944 KB)

## Data Availability

Data were submitted to Metabolights website: https://www.ebi.ac.uk/metabolights/MTBLS12704.
