# Supplementary material for: Exploring salivary metabolites as biomarkers in chronic craniofacial and orofacial pain: a metabolomic analysis
Source: Metabolomics. 2025 Sep 4;21(5):133. doi: 10.1007/s11306-025-02336-x (PMC12411589; doi:10.1007/s11306-025-02336-x)

**Suppplementary figure 1.** Workflow diagram summarizing untargeted vs. targeted analyses. The figure was created with BioRender.com.


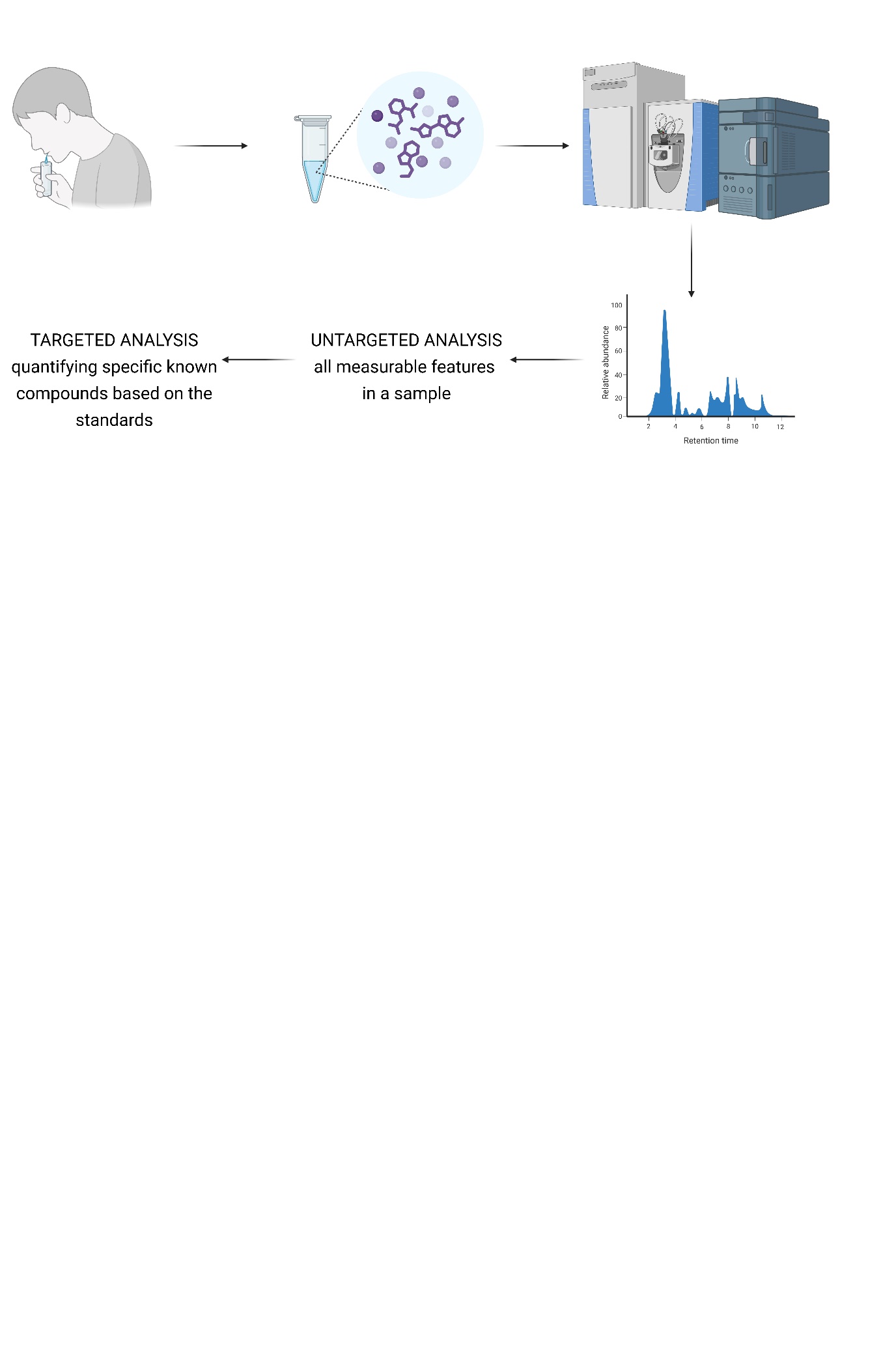


**Supplementary figure 2.** Volcano plot combines results from Fold Change (FC) Analysis and T-tests. A sex-stratified analysis comparing control vs. pain groups in men (A) and women (B) was performed separately.

A


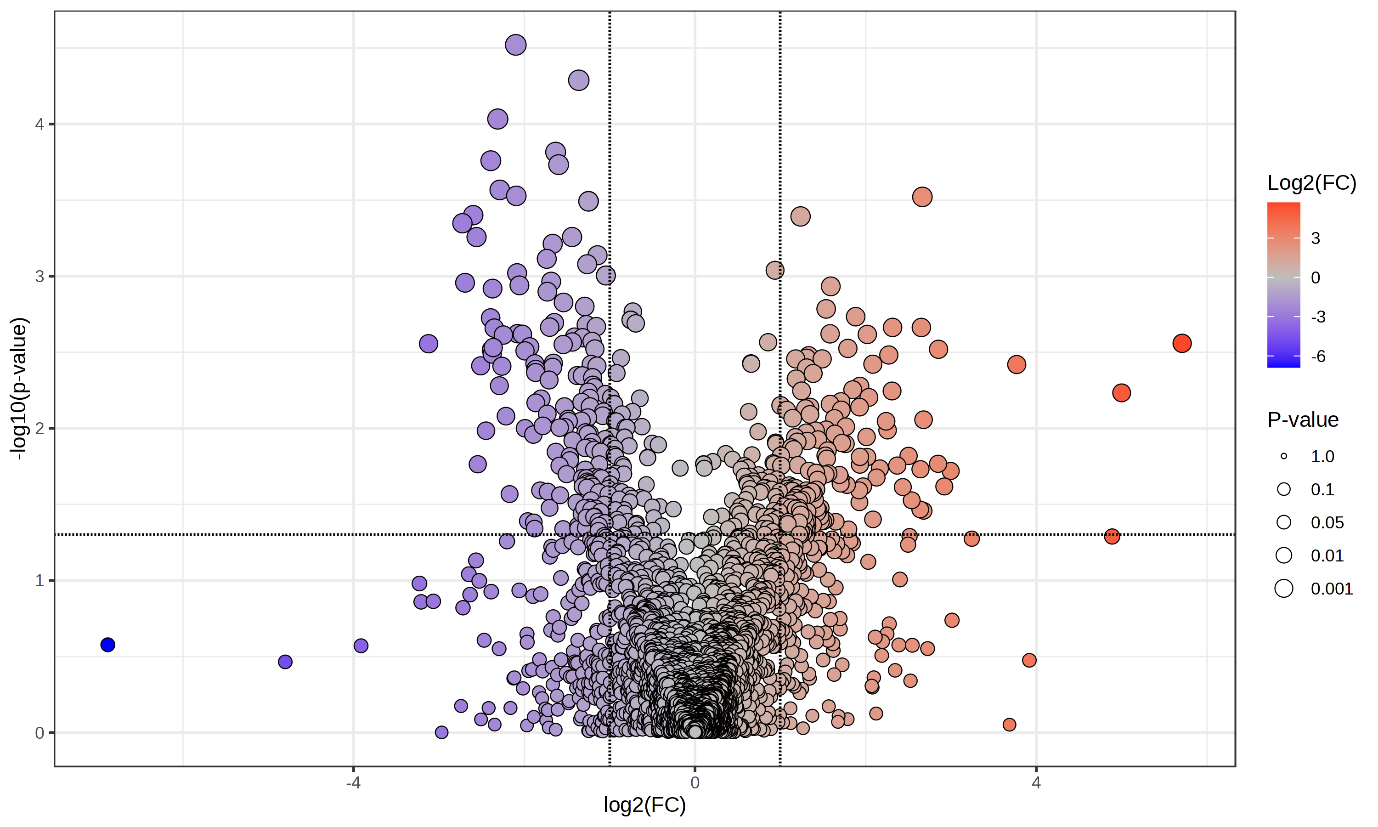


B


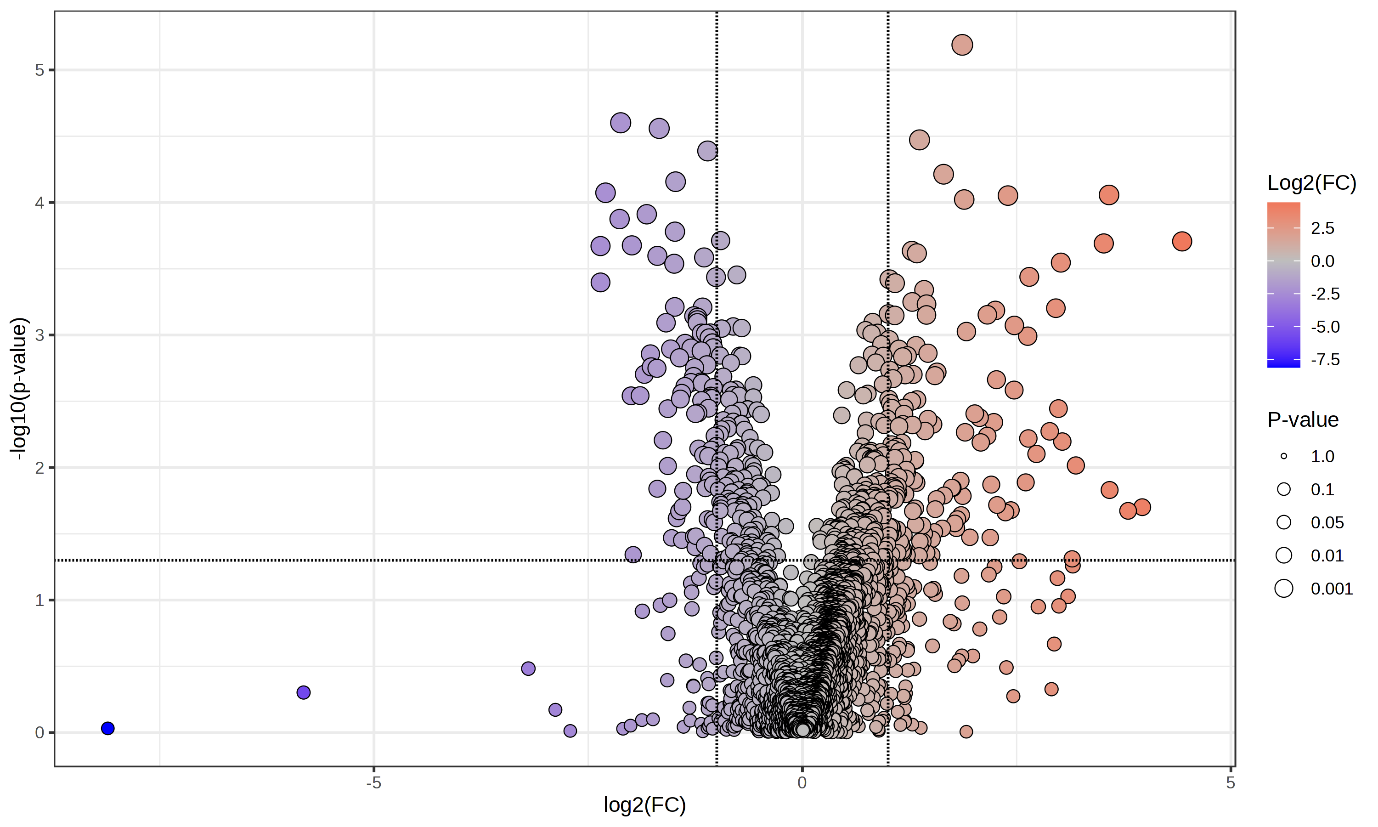

Supplement: Supplementary file 2 — Supplementary Material 2 (944 KB) [file 11306_2025_2336_MOESM2_ESM.docx]
